# CRLF1 Is a Key Regulator in the Ligamentum Flavum Hypertrophy

**DOI:** 10.3389/fcell.2020.00858

**Published:** 2020-09-18

**Authors:** Zhenyu Zheng, Xiang Ao, Peng Li, Zhengnan Lian, Tao Jiang, Zhongmin Zhang, Liang Wang

**Affiliations:** ^1^Department of Orthopedics, The Third Affiliated Hospital, Southern Medical University, Guangzhou, China; ^2^Academy of Orthopedics, Guangzhou, China; ^3^Division of Spine Surgery, Department of Orthopadics, Nanfang Hospital, Southern Medical University, Guangzhou, China

**Keywords:** ligamentum flavum hypertrophy, CRLF1, fibrosis, proteomics analysis, mouse model

## Abstract

Hypertrophy of the ligamentum flavum (HLF) is one of the common causes of lumbar spinal stenosis (LSS). The key molecules and mechanisms responsible for HLF remain unclear. Here, we used an integrated transcriptome and proteomics analysis of human ligamentum flavum (LF), and subsequent immunohistochemistry and real-time PCR assays, to show upregulation of CRLF1 to be the dominant response to HLF. TGF-β1 significantly increased mRNA expression of CRLF1 through SMAD3 pathway. CRLF1 enhanced LF fibrosis via ERK signaling pathway at the post-transcriptional level and was required for the pro-fibrotic effect of TGF-β1. Knockdown of CRLF1 was shown here to reduce fibrosis caused by inflammatory cytokines and mechanical stress. Furthermore, we found that bipedal standing posture can cause HLF and upregulation of CRLF1 expression in mice LF. Overexpression of CRLF1 was indicated to cause HLF *in vivo*, whereas CRLF1 knockdown impeded the formation of HLF in bipedal standing mice. These results revealed a crucial role of CRLF1 in LF hypertrophy. We propose that inhibition of CRLF1 is a potential therapeutic strategy to treat HLF.

## Introduction

Lumbar spinal stenosis (LSS) is the most common spinal disorder in elderly patients (Backstrom et al., 2011; Altun and Yuksel, 2017). The prevalence of symptomatic LSS is 9.3% (Ishimoto et al., 2012). Hypertrophy of the ligamentum flavum (HLF) is a major contributor to LSS (Yoshida et al., 1992; Saint-Louis, 2001), which may cause compression of the nerve root or cauda equina (Beamer et al., 1973; Munns et al., 2015). In HLF, elastic fibers are replaced by excessive collagen tissue, resulting in fibrosis, which causes ligamentum flavum hypertrophy (LFH) (Sairyo et al., 2005, 2007; Kosaka et al., 2007).

Previous studies have suggested that mechanical stress was the initial cause of HLF (Fukuyama et al., 1995; Nakatani et al., 2002) and could induce several cytokines espression in fibroblasts, such as transforming growth factor (TGF-β1) and IL-6 (Koyama and Aizawa, 2002; Nakatani et al., 2002; Jacobs et al., 2014). Although these cytokines have been reported to induce fibrosis in a variety of soft tissues (Mozaffarian et al., 2008; Diaz et al., 2009; Gonzalez et al., 2016; Li et al., 2019), the key cytokines and precise mechanism of the pathology of HLF has not been fully elucidated. To identify genes that may contribute to the pathogenesis of HLF, we performed isobaric tags for relative and absolute quantification (iTRAQ) analysis and integrated proteome and transcriptome data. Our results revealed significantly increased expression of cytokine receptor-like factor 1 (CRLF1).

CRLF1 is a secreted protein that belongs to the cytokine receptor family. It is generally believed that CRLF-1 is secreted as a heterodimer bound to cardiotrophin like cytokine factor 1 (CLCF1), a member of the IL-6 family of cytokines (Elson et al., 2000). However, recent studies found that CRLF1 may also be important in the absence of this binding partner (Looyenga et al., 2013). In addition to cold sweat syndrome (Knappskog et al., 2003) and Crisponi syndrome (Crisponi et al., 2007), mutations in the CRLF1 gene can also cause facial muscle atrophy, scoliosis and craniofacial deformity (Yamazaki et al., 2010). Thus, CRLF1 may be involved in extracellular matrix (ECM) synthesis.

At present, it has been found that many pro-fibrotic factors can promote the secretion of CRLF1. Fleissig found that CRLF-1 can be upregulated in deciduous periodontal ligament fibroblasts by mechanical stress (Fleissig et al., 2018). Elson found that CRLF-1 can be upregulated in some fibroblast cells by tumor necrosis factor-α (TNF-α), interleukin-6 (IL-6), and interferon-γ (Elson et al., 1998). Tsuritani reported that the secretion of CRLF1 was regulated by TGF-β1 in chondrocytes (Tsuritani et al., 2010). However, the functions of CRLF1 in the pathogenesis of HLF are unknown. In this study, we investigated the regulatory mechanism of CRLF-1 in HLF and explored the role of CRLF-1 *in vivo*.

## Materials and Methods

### Human LF Sample Collection

This study was approved by the Institutional Ethics Review Committee of the Third Affiliated Hospital of Southern Medical University. Informed consent was obtained from each patient before surgery. Ligamentum flavum (LF) tissues were collected from the anatomical region (L4/5). Twenty LSS patients with LF thickening (>4 mm) were assigned to the HLF group, twenty lumbar disc herniation patients with LF <3 mm were assigned to the non-HLF group. Patient information is shown in Supplementary Table S1.

### Proteomic Profile by Mass Spectrometry

Ligamentum flavum samples from three patients diagnosed LSS with HLF were defined as the HLF group. LF samples from three patients diagnosed lumbar disc herniation with normal LF served as the non-HLF group. Protein extraction and iTRAQ were established according to previous reports (Wang et al., 2017). In short, LF tissues (200 mg) were homogenized in liquid nitrogen and lysed in 250 μl of RIPA lysis buffer. Then, the sample was sonicated on ice and centrifuged at 12,000 × *g* for 20 min at 4°C, and the middle layer of liquid was collected. Proteins were digested with trypsin (Promega, United States) at 37°C at a ratio of 1:50 overnight. iTRAQ labeling was performed according to the manufacturer’s protocol (Applied Biosystems, Sciex). Each sample was labeled separately with two of the eight available tags. All labeled peptides were pooled together. The Ultimate 3000HPLC system (Dionex, United States) was used for High-pH fractionation. The UV detector was set at 214/280 nm, and fractions were collected every 1 min. In total, 20 fractions were pooled and dried by vacuum centrifuge for subsequent nano-reversed phase liquid chromatography (nano-LC) fractionation. For RPLC-MS/MS Analysis, each fraction was resuspended in loading buffer (0.1% formic acid, 2% acetonitrile) and separated using an Ultimate 3000 nano-LC system equipped with a C18 reverse phase column. The peptides were separated using the following parameters: (1) mobile phase A: 0.1% formic acid, 5% acetonitrile, dissolved in water; (2) mobile phase B: 0.1% formic acid, 95% acetonitrile; (3) flow rate: 300 nl/min; (4) gradient: B-phase increased from 5 to 40%, 70 min. Then, samples was subject to Q Exactive (Thermo Fisher) in an information dependent acquisition mode. MS spectra were acquired across the mass range of 400–1250 m/z in high resolution mode (>30,000) using 250 ms accumulation time per spectrum. A maximum of 20 precursors per cycle were chosen for fragmentation from each MS spectrum with100 ms minimum accumulation time for each precursor and dynamic exclusion for 20 s. Tandem mass spectra were recorded in high sensitivity mode (resolution >15,000) with rolling collision energy on and iTRAQ reagent collision energy adjustment on. The peptide data were analyzed using Protein Pilot Software 4.0 (AB SCIEX, CA, United States). Only proteins with at least one unique peptide and unused value more than 1.3 were considered for further analysis.

### Integrated Analysis of Transcriptomic and Proteomic Data

The peptide data from iTRAQ were analyzed using Protein Pilot Software 4.0 (AB SCIEX, CA, United States). For data analysis, Log2 FC (fold change) >1.5, *p*-value < 0.05, and CV < 0.5 were used to categorize proteins as significantly changed. The gene expression profiles of GSE113212 were downloaded from Gene Expression Omnibus (GEO) datasets^1^. The differentially expressed genes were analyzed with the Limma package (version 3.34.8) using the R language. Log2 FC ≥ 2 and adjusted *p*-value <0.01 were considered as cut-off criteria. The proteins co-upregulated in two datasets were found using Excel software.

### Histological Studies

The LF samples collected from mice or humans were fixed, decalcified, dehydrated, embedded in paraffin blocks, and cut into 4-μm-thick sections. The sections were deparaffinized, rehydrated and subjected to H&E (Sigma, United States) or EVG (Leagene, China) staining according to the manufacturer’s instructions. Quantitative analyses of the LF areas and the ratio of elastic fibers to collagen fibers of mice LF were achieved using ImageJ software (NIH, United States).

### Immunofluorescence and Immunohistochemistry Analysis

After deparaffinization and rehydration, sections were incubated in citrate buffer for 16 h at 60°C to retrieve the antigen. Sections for immunohistochemistry (IHC) were treated with 3% hydrogen peroxide for 15 min and then blocked with 1% goat serum for 1 h. Then sections were incubated with primary antibodies against CRLF1 (1:100, ab211438, Abcam), GFP (1:100, EPR14104, Abcam), and a-SMA (1:100, ab7817, Abcam) for IHC, and a-SMA (1:100, ab7817, Abcam), CRLF1 (1:100, ab211438, Abcam) for Immunofluorescence (IF). Species-matched secondary antibody was used. Then, DAB (Beyotime, China) and haematoxylin (Beyotime, China) were used for IHC, and DAPI was used for IF.

### Culture of LF Cells

To extract of human LF cells, human normal LF specimens were obtained from patients who underwent posterior decompression surgery. The samples were cut into small pieces and digested for 1 h at 37°C using 0.2% type I collagenase (17100017, Gibco). Then washed with Dulbecco’s modified Eagle’s medium (DMEM, Gibco) and incubated with high-glucose DMEM with 20% fetal bovine serum (FBS, 10099141C, Gibco).

To extract mouse LF cells, mice were killed after anesthesia, and the L4–6 segments were obtained under aseptic conditions. The vertebral body was cut off, the nerves and the dural tissue were removed, and the LF was cut off with the aid of an operating microscope (Olympus SZX 16, Japan) and microsurgical instruments. The incubation conditions were the same as those for the human LF cells. All experiments were carried out at cell passage (<P5). All cells were cultured in serum-free media for 16 h before treatment. The ERK and SMAD3 pathway inhibitors used in this study were U0126 (KeyGEN, China) and SIS3 (MedChem Express, United States).

### Cell Proliferation and Scratch Wound Assay

Cell viability was quantified using 3-(4,5-dimethyl-2-thiazolyl)-2,5-diphenyl-2-H-tetrazolium bromide (MTT, Solarbio) according to the manufacturer’s instructions. The migratory ability was measured using scratch wound assays. A scratch was made in the confluent monolayers of cells with a sterile pipette tip. Cells were subjected to mature recombinant human CRLF1 (mammalian-expressed) (IC8303, Immuno Clone) or recombinant human TGF-β1 (10804-HNAC-5, Sino Biological) for 48 h. The scratch healing rate was quantified by measuring the percentage change in the scratch area.

### RT-qPCR

Total RNA was extracted from LF tissue or cells with TRIzol (Thermo Fisher Scientific). cDNA was synthesized from this RNA using PrimeScript RT Master Mix (Takara Biotechnology Co. Ltd., Shija, Japan). Real-time PCRs were performed with TB Green Premix Ex Taq according to the manufacturer’s instructions (Takara). The 2^–ΔΔCt^ method was used to calculate the relative gene expression fold change. The primers purchased from Shanghai Biological Engineering are listed in Supplementary Table S2.

### Western Blot Analysis

Cell lysates from the experimental and control groups were collected and prepared for western blot. Primary antibodies against CRLF1 (1:1000, ab211438, Abcam), collagen I(1:1000, AF7001, Affinity), collagen III (1:1000, GTX111643, Gene Tex), MMP2 (1:1000, AF5300, Affinity), a-SMA (1:1000, ab7817, Abcam) and GAPDH (1:5000, RM2002, Rayantibody) were incubated overnight at 4°C followed by incubation with the appropriate secondary antibodies: goat anti-rabbit IgG (H+L)-HRP (1:3000, RM3002, Rayantibody) and goat anti-mouse IgG (H+L)-HRP (1:3000, RM30011, Rayantibody). Proteins were detected using an enhanced chemiluminescence kit (KF005, Affinity).

### RNA Interference

CRLF1-targeting small interfering RNAs (siRNAs) were synthesized by Shanghai Biological Engineering. The sequences are shown in Supplementary Table S3. The siRNAs were transfected with the RFect Transfection Reagent (BIOG-11014, BAIDAI Biotech, China) at a final concentration of 50 nM according to the manufacturer’s instructions. After 48 h of transfection, the cells were then cultured in serum-free media for 16 h and then split into different groups that were subjected to different treatment.

### Application of Mechanical Stretch on Cells

The cell stretching device was a FX5000 Tension (Flexcell International Corp., United States). The LF cells were seeded on six-well Bioflex plates (BF-3001P, BioFlex, United States). Six hours after seeding, cells were transfected with siRNA. After 48 h of transfection, the cells were then cultured in serum-free media for 16 h and then subjected to cyclic stretch with cycles of 10 s of 20% elongation and 10 s of relaxation for 24 h. The control group were subjected to the same conditions without mechanical stretch.

### Adeno-Associated Virus (AAV) Gene Transfer Unit

The AAV vector can provide several months of transgene expression with a reduced immune response to the vector itself (Nixon et al., 2012). AAV2-mediated siCRLF1, CRLF1 overexpression and vectors with enhanced GFP were produced by Hanbio Biotechnology (Shanghai, China).

### Animal Experiments

All animal experimental protocols were approved by the Ethics Committee for Animal Research of Southern Medical University. All mice purchased from the Experimental Animal Center of Southern Medical University were 8-week-old C57BL/6 male mice. We established the mouse HLF model as previously described (Ao et al., 2019) by taking advantage of the hydrophobia of mice (Pistell and Ingram, 2010) to induce them to adopt a bipedal standing posture for 6 h a day with an interval of 2 h of free activity. To verify the validity of the mouse HLF model, 16 mice were randomly assigned to the control and the bipedal standing group with 8 mice in each group. After 10 weeks of modeling, the LF area, the ratio of elastic fibers to collagen fibers and expression of CRLF1 were quantified. To verify the validity of the transfection efficiency of AAV2 *in vivo*, 8 mice were randomly assigned to the control and the AAV2-GFP group with 4 mice in each group. 4 weeks after AAV2-GFP injection, IHC was performed to observe the GFP expression in LF. To verify the role of CRLF1 *in vivo*, AAV2-CRLF1 was used. A total of 48, 8-week-old mice were randomly assigned to the control group, AAV2-vector group, AAV2-CRLF1 group, AAV2-vector + bipedal standing group, AAV-CRLF1 + bipedal standing group, and AAV-siCRLF1 + bipedal standing group (8 mice in each group). AAV injections were performed under anesthesia with intraperitoneal chloral hydrate. For each case, a longitudinal skin incision was made over the lumbar spine, and L5/6 LF were exposed by removing dorsal paravertebral muscles from the spinous processes and laminae with the aid of an operating microscope. The viral solution (3 μl) (1 × 10^12^ vg/ml) was injected into the left LF with a microinjector (NF36BV 36GA, NanoFil, United States). The dorsal paravertebral muscles and fasciae were repaired and the wound was closed. Animals were allowed to recover for 3 weeks before the experiments were carried out. After 10 weeks of modeling, mice were euthanized and intact L5/6 vertebrae were harvested. Histological analyses were performed to measure the area of the LF.

### Statistical Analyses

Each experiment was repeated at least three times. All data from *in vivo* and in vitro experiments presented as the mean ± SD. Statistical analyses of data were performed using SPSS 20.0 (SPSS, Chicago, IL, United States). The results were compared by performing *t*-tests or one-way analysis of variance (ANOVA). *p* < 0.05 was considered to be statistically significant.

## Results

### Identification of Differentially Expressed Genes

**FIGURE 1:**
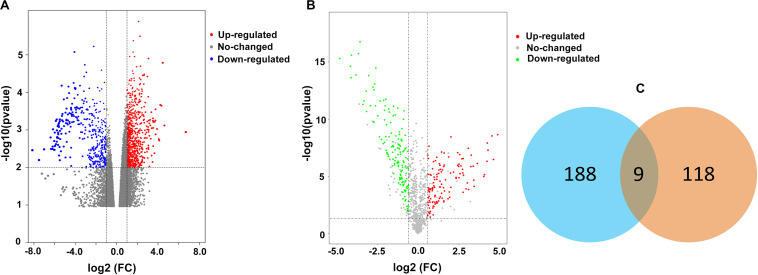
Differentially expressed proteins in HLF. **(A)** Volcano plot of RNA-seq data. The vertical lines correspond to 2.0-fold increase and decrease, whereas the horizontal line represents a *p*-value of 0.01. **(B)** Volcano plot of iTRAQ data. The vertical lines correspond to 1.5-fold increase and decrease, whereas the horizontal line represents a *p*-value of 0.05. **(C)** The two datasets showed an overlap of nine genes.

A total of 197 upregulated genes were identified from an RNAseq analysis (Figure 1A, Supplementary Table S4). iTRAQ analysis identified 127 upregulated proteins (Figure 1B, Supplementary Table S5). An integrated analysis of these two datasets identified nine significantly upregulated proteins in HLF tissues (Figure 1C and Table 1), and CRLF1 was the most highly upregulated in HLF.

**TABLE 1:** Differentially expressed proteins in HLF tissues compared with normal LF.

**Gene name**	**Fold change**	***P-*value**
CRLF1	23.686163	3.00E-09
CILP	16.881399	1.02E-05
THBS1	9.7086238	1.42E-06
SERPINE2	8.7795014	0.000141
ACAN	7.8143086	0.000121
HAPLN1	5.8259634	8.70E-07
FGFBP2	4.1008459	3.47E-09
CRTAC1	3.2614812	2.09E-05
COL2A1	1.7246353	0.000884

### High Expression of CRLF1 in HLF Myofibroblasts

**FIGURE 2:**
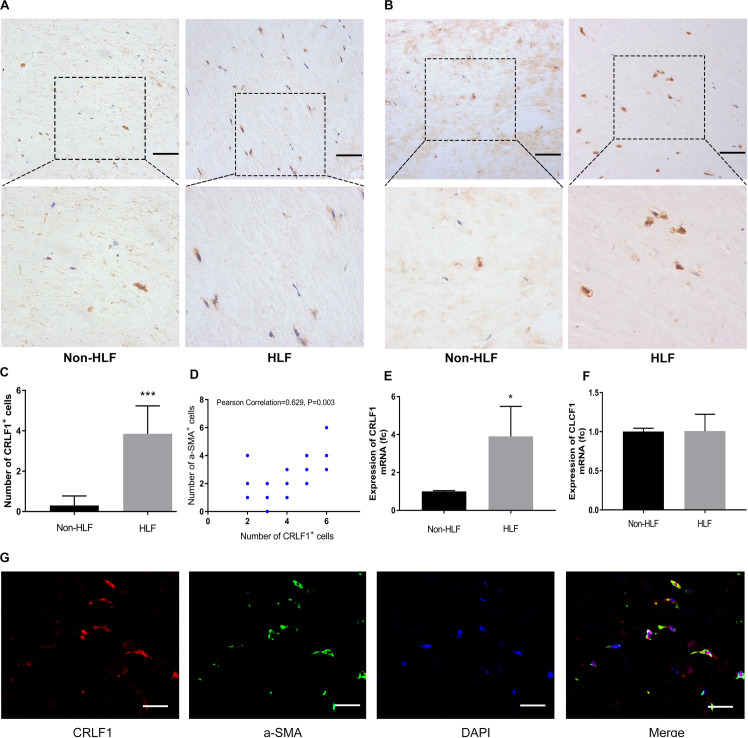
CRLF1 is upregulated in HLF. Representative photographs of IHC staining of CRLF1 **(A)** and a-SMA **(B)**. Scale bars: 50 μm. **(C)** Quantitative analysis of CRLF1-positive cells (*n* = 20, ***p < 0.001). **(D)** Correlation between the number of CRLF1- and a-SMA- positive cells (*n* = 20, *R* = 0.629, *p* = 0.003). Comparison of CRLF1 mRNA **(E)** and CLCF1 mRNA **(F)** expression in the HLF and non-HLF samples (*n* = 4, *p < 0.05). **(G)** Double immunofluorescence staining for CRLF1 and a-SMA. Nuclei were stained with DAPI. Scale bars: 20 μm. The data represent the mean ± SD.

Considering the widely different expression levels of CRLF1 in multiomics analysis, we used IHC to verify the results and found that CRLF1 was upregulated in all HLF samples (Figures 2A,C). a-SMA which expressed in myofibroblasts is a feature of fibrosis. In this study, we found increased myofibroblasts in HLF (Figure 2B), and the number of CRLF1-positive cells was positively correlated with the number of myofibroblasts (Figure 2D). In addition, RT-qPCR analysis showed a marked increase in CRLF1 mRNA in the HLF group (Figure 2E), whereas CLCF1 mRNA (Figure 2F) was unchanged. Furthermore, double IF staining showed good co-expression of CRLF1 and a-SMA in HLFs (Figure 2G), which suggests that CRLF1 is produced by myofibroblasts. Taken together, these findings indicated that LF myofibroblasts-derived CRLF1 may act as an independent regulator which contribute to the pathological process of LFH.

### Effect of CRLF1 on Fibrosis of LF Cells

**FIGURE 3:**
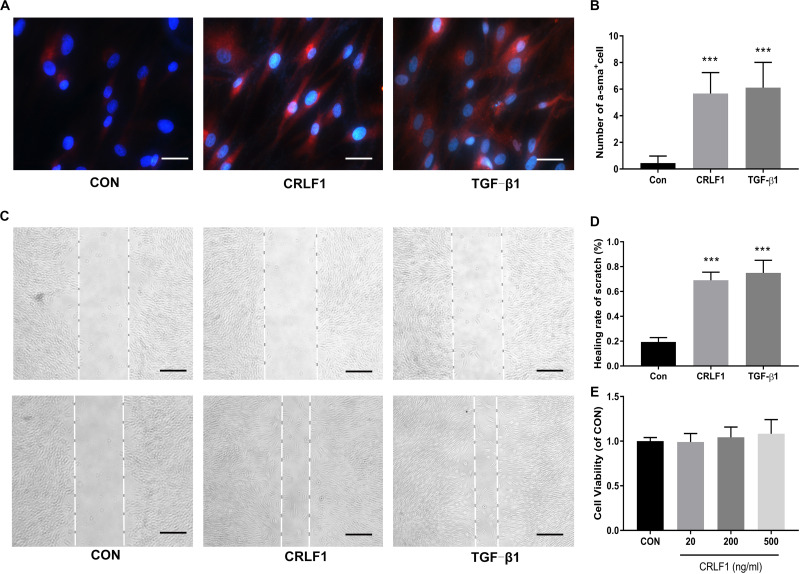
CRLF1 causes a phenotypic transformation and migratory ability in LF cells. **(A)** Cells treated with CRLF1 (500 ng/ml) and TGF-β1 (5 ng/ml) for 48 h and then IF stained to assess the expression of a-SMA. Scale bars: 20 μm. **(B)** Quantitative analysis of a-SMA-positive cells (*n* = 3, ***p < 0.001). **(C)** Migratory ability was evaluated using a scratch assay after 48 h treatment with CRLF1 (500 ng/ml) and TGF-β1 (5 ng/ml). Scale bars: 100 μm. **(D)** Quantitative analysis of the scratch assay (*n* = 3, ***p < 0.001). **(E)** Cell viability was assessed using MTT assay (*n* = 3). The data represent the mean ± SD.

TGF-β1 is known to induce fibrosis in LF cells, which is involved in the formation of HLF (Nakatani et al., 2002). To investigate the effects of CRLF1 on the LF fibrosis, we first induced fibrosis in primary LF cells with CRLF1 and TGF-β1. IF-stained cells showed that CRLF1 and TGF-β1 obviously promoted the trans-differentiation of LF cells into myofibroblasts (Figures 3A,B). Then, scratch assays were performed to assess the effect of CRLF1 on cell motility. Compared with the control group, the TGF-β1 group significantly promoted scratch closure after 48 h of treatment, and cells from the CRLF1 group showed migration abilities similar to those in the TGF-β1 group (Figures 3C,D). We next performed MTT assay to investigate the potential toxicity on cell proliferation. No significant difference was observed between the different groups after 48 h of treatment (Figure 3E), which indicated that stimulations with CRLF1, even at a concentration of 500 ng/mL, had no toxicity on the growth of LF cells.

**FIGURE 4:**
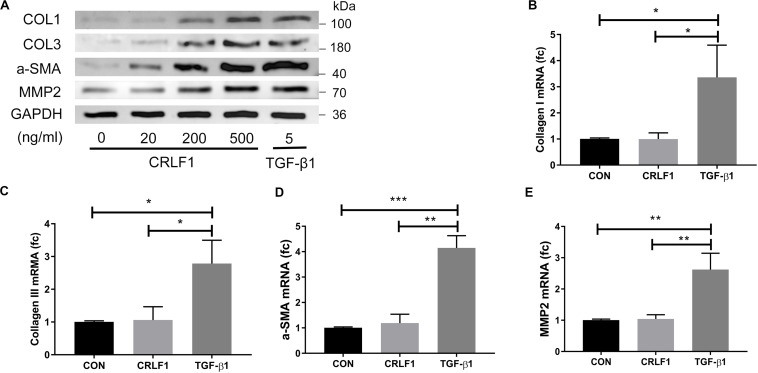
CRLF1 stimulates the cell matrix at the post-transcriptional level. LF cells were exposed to CRLF1 for 24 h. **(A)** Cell lysates were obtained for Western blot analysis. **(B–E)** Fibrosis markers mRNA were upregulated in response to TGF-β1 but not to CRLF1 (*n* = 3, *p < 0.05, **p < 0.01, ***p < 0.001). The data represent the mean ± SD.

Subsequently, RT-qPCR and Western blot were performed to analyze the expression of fibrosis markers after treatment of CRLF1 and TGF-β1 for 24 h. The up-regulation of the fibrosis markers were observed at protein levels (Figure 4A). To our surprise, the effects of CRLF1 on the mRNAs were negligible, whereas TGF-β1-driven transcriptional regulation was profound in LF cells (Figures 4B–E). Taken together, these results elucidate that CRLF1-induced fibrosis were concluded to occur mainly at the post-transcriptional level.

### TGF-β1 Regulates CRLF1 Expression Through the SMAD3 Pathway

**FIGURE 5:**
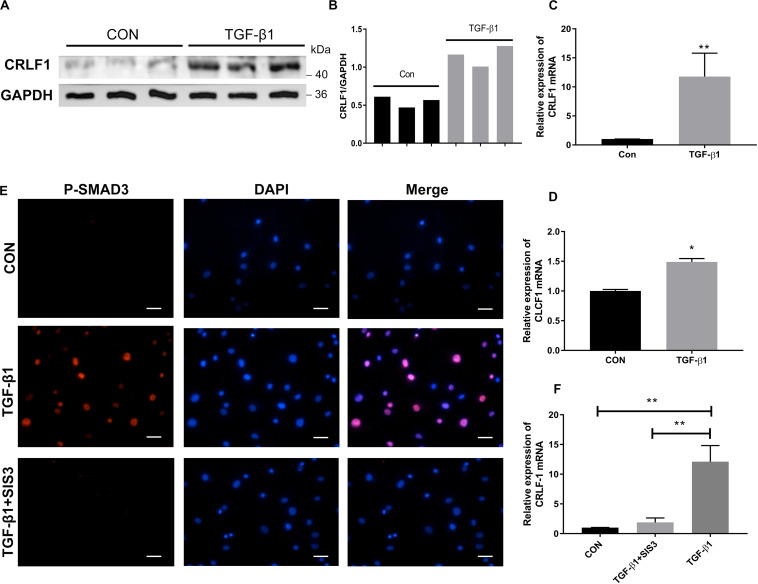
The regulation of CRLF1 by TGF-β1 is SMAD3-dependent. **(A)** CRLF1 expression in the LF cells with or without TGF-β1 treatment (5 ng/ml). **(B)** Quantitative analysis of gray values. **(C)** CRLF1 mRNA expression increased significantly after 24 h of treatment with TGF-β1 (11.8 ± 2.3-fold change) (*n* = 3, **p < 0.01). **(D)** CLCF1 mRNA expression increased after 24 h of treatment with TGF-β1 (1.49 ± 0.03-fold change) (*n* = 3, *p < 0.05). **(E)** Nuclear translocation of SMAD3 by TGF-β1 (5 ng/ml, 1 h), and inhibition of the translocation by SIS3 (10 μM) in LF cells. **(F)** SIS3 inhibited the expression of CRLF1 mRNA promoted by TGF-β1 (5 ng/ml, 24 h) (*n* = 3, **p < 0.01). Scale bars: 20 μm. The data represent the mean ± SD.

Because CRLF1 expression can be upregulated by TGF-β1 in chondrogenic cells (Tsuritani et al., 2010), we set out to explore the relationship between CRLF1 and TGF-β1 in LF cells. Western blot analysis showed that TGF-β1 significantly promoted the protein production of CRLF1 (Figures 5A,B). RT-qPCR showed that the expression of CRLF1 mRNA induced by TGF-β1 increased to 11.8 ± 2.3 times (Figure 5C). In contrast, TGF-β1 only increased CLCF1 mRNA synthesis to 1.49 ± 0.03 times (Figure 5D). Thus, TGF-β1 is proposed to mainly promotes the CRLF1 expression. To further investigate the regulation mechanism of TGF-β1 on CRLF1, SIS3, a SMAD3 inhibitor was used. Western blot and RT-qPCR analysis found that SIS3 suppressed the SMAD3 nuclear translocation (Figure 5E) and rescued the upregulation of CRLF1 mRNA induced by TGF-β1 (Figure 5F). These data suggested that TGF-β1 regulates CRLF1 expression through the SMAD3 pathway.

### CRLF1 Mediates TGF-β1 Induced Fibrosis Through the ERK Signaling Pathway

**FIGURE 6:**
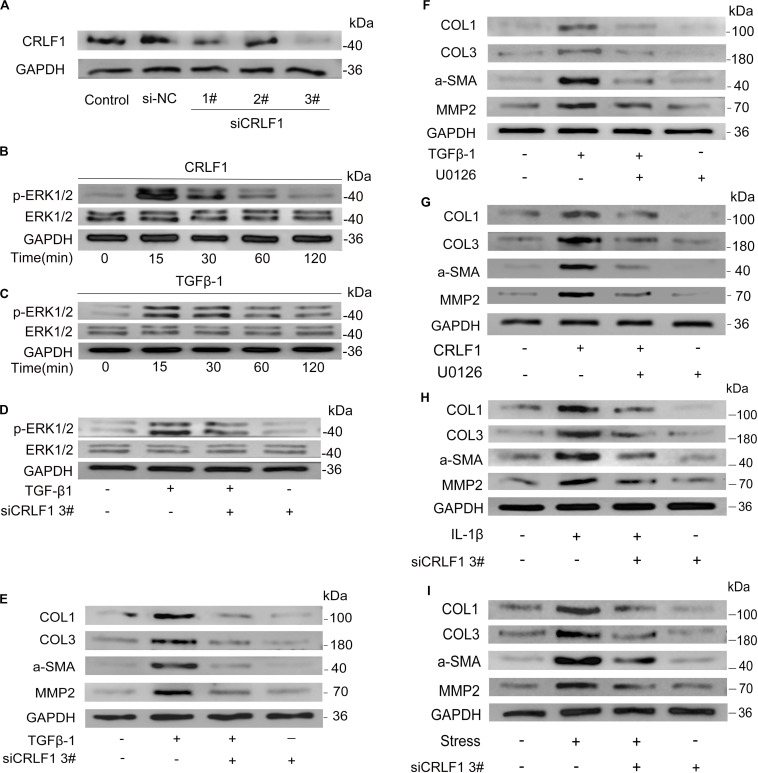
CRLF1 plays a crucial role in LF fibrosis. **(A)** CRLF1 expression induced by TGF-β1 in LF cells transfected with siRNAs. **(B,C)** ERK signaling pathway activation by CRLF1 and TGF-β1. **(D)** siCRLF1 attenuated the ERK phosphorylation induced by TGF-β1. **(E)** siCRLF1 reduced the fibrosis markers expression induced by TGF-β1. **(F,G)** Inhibition of the ERK pathway attenuated the CRLF1- and TGF-β1-induced expressions of fibrosis markers in LF cells. **(H)** siCRLF1 significantly reduced the pro-fibrotic effect of IL-1β (20 ng/ml). **(I)** siCRLF1 significantly reduced the pro-fibrotic effect of mechanical stretching forces.

ERK signaling is an important pathway for fibrosis (Davis and Molkentin, 2014). To investigate the possible involvement of CRLF1-ERK pathway in TGF-β1-induced fibrosis, CRLF1 specific siRNA (Figure 6A) and U0126 were used. Westen blot showed that both CRLF1 and TGF-β1 activated the ERK pathway in LF cells (Figures 6B,C) and that the response was greatest at 15 min. In the presence of siCRLF1, both activation of ERK and expression of fibrosis markers induced by TGF-β1 were reduced (Figures 6D,E). Upon pretreatment with U0126 (20 μM) for 30 min, both CRLF1- and TGF-β1-induced expression of fibrosis markers was significantly attenuated (Figures 6F,G).

To further delineate the key role of CRLF1 in the formation of HLF, LF cells were incubated with IL-1β or subjected to mechanical stretching forces in the presence or absence of siCRLF1. Western blot analysis revealed that inhibition of CRLF1 significantly reduced the pro-fibrotic effect of these stimuli (Figures 6H,I).

### CRLF1 Plays a Critical Role in the Formation of HLF *in vivo*

**FIGURE 7:**
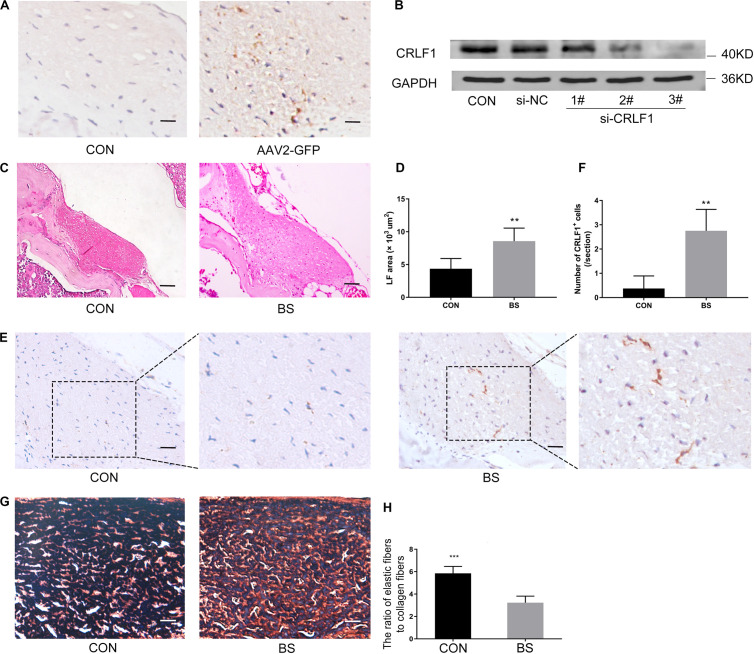
Bipedal standing mice can simulate the pathological changes of human HLF. **(A)** The AAV2-GFP was successfully transfected into the LF. scale bars: 5 μm. **(B)** Mouse LF cells transfected with siCRLF1 3# showed a marked reduction of CRLF1 after 24 h of TGF-β1 (5 ng/ml) treatment. **(C)** The LF area in bipedal standing mice was increased compared to that in control mice. Scale bars: 20 μm. **(D)** Quantitative analysis of the LF area (*n* = 8, **p < 0.01). **(E)** IHC staining of CRLF1 in mice LF. Scale bars: 10 μm. **(F)** Quantitative analysis of the number of CRLF1-positive cells (*n* = 8, **p < 0.01). **(G)** Representative images of EVG-stained LF. Scale bars: 5 μm. **(H)** Quantitative analyses of the ratio of elastic fibers area to collagen fibers area (*n* = 8, ***p < 0.001). CON = control group; BS = 10-weeks of bipedal standing group. The data represent the mean ± SD.

**FIGURE 8:**
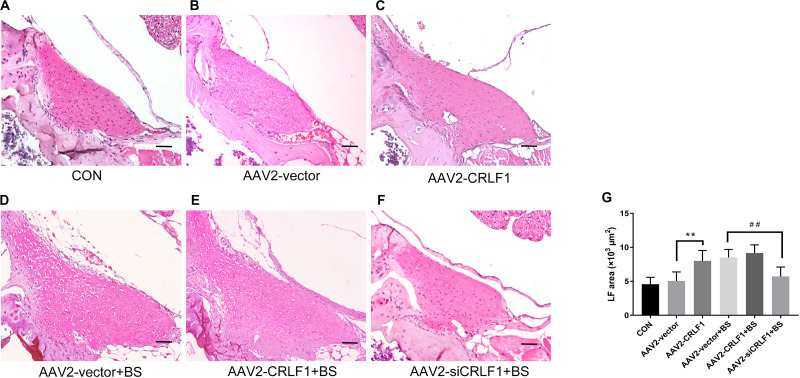
CRLF1 plays a crucial role in the formation of HLF *in vivo*. **(A–F)** Representative images of H&E-stained LF. Scale bars: 20 μm. **(G)** Quantitative analyses of the LF area (*n* = 8, **p < 0.01 versus the AAV-vector group, ^##^*p* < 0.01 versus the AAV-vector+BS group). CON = control group; BS = 10-weeks of bipedal standing group. The data represent the mean ± SD.

The AAV vector system is a promising delivery vehicle that has been shown to be nonpathogenic and noncytotoxic (Daly, 2004; Zhu et al., 2006). First, we used AAV2-GFP to verify the transfection efficiency of the virus. Results showed that the AAV2 vector effectively transfected the GFP gene into the LF of mice 4 weeks after injection (Figure 7A). The inhibitory effect of CRLF1-specific siRNAs in mouse LF cells is shown in Figure 7B. siRNA 3# sequence was used to construct AAV2-siCRLF1. Then, bipedal standing mice were used to make the HLF model. As shown in Figures 7C–F, the average LF area and the number of CRLF1-positive cells in the 10-week bipedal standing mice increased significantly. In addition, the ratio of elastic fibers to collagen fibers decreased (Figures 7G,H). These results were consistent with the human data (Figure 2A) and previouse studies (Zhong et al., 2011), demonstrating the high validity of the model. To further illustrate the role of CRLF1 on the LF, both AAV2-CRLF1 and AAV2-siCRLF1 were used. No significant difference was observed in the LF area between the control and AAV2-vector group (Figures 8A,B) indicating that the administration of the AAV2 vector did not promote HLF. Compared with the AAV2 vector group, the AAV2-CRLF1 group obviously increased HLF formation (Figures 8B,C), whereas AAV2-siCRLF1 inhibited LFH formation in bipedal standing mice (Figures 8D,F). Measures showing a statistically significant change with LF area indicated by ^∗∗^*p* < 0.01 and ^##^*p* < 0.01, respectively (Figure 8G). Taken together, these data suggested that inhibition of CRLF1 could suppress the LFH formation in bipedal standing mouse model.

## Discussion

Although aging and mechanical stress both of which are external factors are considered to be main factors involved in HLF (Sairyo et al., 2005; Altinkaya et al., 2011; Kolte et al., 2015), the key internal molecular mechanisms remain unknown. This study investigated the common internal pathways up regulated by the external factors and explored it’s mechanisms. Herein, we used proteomics to analyze the different protein expression in HLF and normal LF. The two groups are age-matched. So it can be proposed that the formation of HLF is mechanical stress related in our data. In addition, the GEO dataset was used to make the transcriptional analysis. The profile investigated the gene expression compared the hypertrophic ligament of the elderly with non-hypertrophic ligament of the young. So, it can be proposed that the formation of HLF is age related in this profile. Integration of two data sets will help us to find out the key internal joint molecules in the formation of HLF. Fortunately, we found an overlap of nine genes, among which CRLF1 expression difference is the most obvious.

It has been broadly assumed that the role of CRLF1 is to function as a co-ligand with CLCF1 (Rousseau et al., 2006; Herholz et al., 2011). However, some studies have indicated an autonomous role for CRLF1 (Looyenga et al., 2013). Present study revealed that although CRLF1 was abundantly expressed in HLF, while CLCF1 was not elevated. Thus, the CRLF1 might be a novel independent indicator for HLF.

While mutations in CRLF1 is associated with cold sweat syndrome and Crisponi syndrome, the craniofacial malformations of these patients suggest that CRLF1 may be important for the formation of the ECM (Knappskog et al., 2003; Dagoneau et al., 2007; Okur et al., 2008; Mcgregor et al., 2010; Yamazaki et al., 2010). Previous research found that CRLF1 induced by a pro-fibrotic stimulus can inhibit the expression of collagen III in the liver (Stefanovic and Stefanovic, 2012). In contrast to previous work, we found that CRLF1 is a pro-fibrotic cytokine for LF cells and increased the trans-differentiation of LF cells into myofibroblasts. One possible reason is that the previous studies only focused on the effect of CRLF1 on transcription level. In the present study, our data showed that the pro-fibrotic effects of CRLF1 are mainly based on the post-transcriptional level, whereas TGF-β1 was found to drive transcriptional regulation in LF cells.

TGF-β1 is the primary factor that drives fibrosis (Meng et al., 2016; Hur et al., 2017), known to promote CRLF1 expression in chondrogenic cells (Tsuritani et al., 2010). However, detailed reviews about the role and regulation mechanism between TGF-β1 and CRLF1 are still absent. This study showed that TGF-β1 can promote CRLF1 expression through SMAD3 pathway in LF cells. Inhibition of CRLF1 can effectively attenuated TGF-β1-induced fibrosis. Inaddition, the pro-fibrosis effect of IL-1β can also be inhibited by siCRLF1. This observation may provide a new clinical insight into the contribution of CRLF1 to the pathophysiological regulation of the development of HLF. Moreover, TGF-β1 can regulate the activity of several signaling molecules, such as SMAD and ERK, which is essential for fibrosis (Akhurst and Hata, 2012; Davis and Molkentin, 2014). In the current study, we found that the profibrotic actions of both TGF-β1 and CRLF1 are mediated, at least in part, via ERK signaling pathway. Taken together, our investigation identified for the first time that CRLF1-ERK pathway is involved in HLF downstream of TGF-β1.

Mechanical stress is the most important external factor in the study of LFH. It has been found that inhibition of TGF-β1 can reduce the pro-fibrosis effect of mechanical stress (Nakatani et al., 2002). TGF-β1 as the main pro-fibrosis factor, was found to promote the expression of CRLF1 mRNA in this study. This is consistent with Fleissig’s studies, which found that mechanical stress can promote CRLF1 mRNA expression in deciduous periodontal ligament fibroblasts (Fleissig et al., 2018). To further elucidate the key role of CRLF1 on fibrosis associated with mechanical stress, siCRLF1 was used. Similarly, inhibition of CRLF1 can reduce the pro-fibrosis effect of mechanical stress. Taken together, our findings may provide a new therapeutic preventive direction for the clinical management of HLF.

Despite the fact that extensive studies have illustrated pathological changes of HLF in vitro, no studies have investigated the potential regulation mechanism of cytokines *in vivo*. In this study, we created a new HLF model in bipedal standing mice. After 10 weeks of bipedal standing, the thickness of the LF, the ratio of elastic fibers to collagen fibers and the expression of CRLF1 increased significantly. This suggested that bipedal standing mice can simulate the pathological changes of HLF in human. Among viral vectors, AAV vectors appear to be a good method for gene therapy strategies (Nixon et al., 2012). In the present study, our data showed that CRLF1 overexpression robustly increased the area of the LF, whereas CRLF1 knockdown prevented HLF formation in bipedal standing mice. Combined with previous studies, we hypothesized that CRLF1 acts as a key regulator of LF tissue homeostasis and excessive CRLF1 promotes pathological remodeling in LF, which lead to HLF.

## Conclusion

**FIGURE 9:**
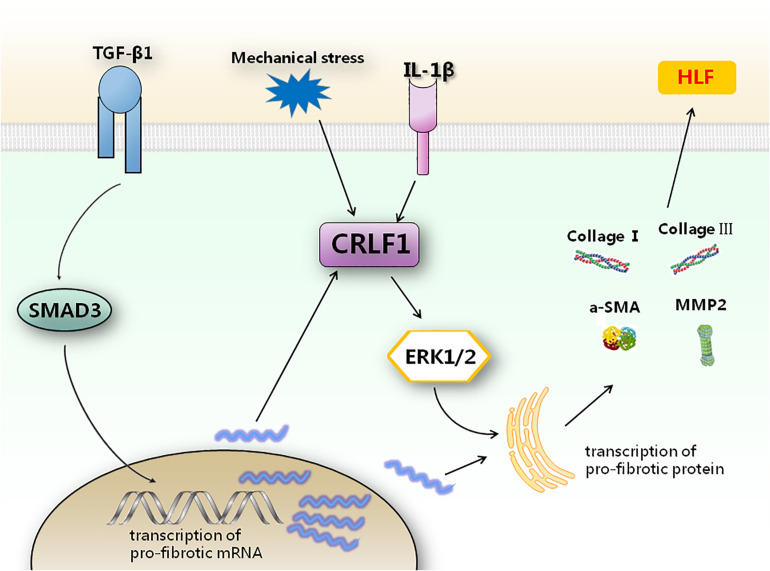
Schematic representation of how CRLF1 promotes HLF formation. TGF-β1 can promote the transcription of pro-fibrotic mRNA. CRLF1 signaling is required to regulate the translation of pro-fibrotic factors by activating the ERK pathway. Inhibition of CRLF1 can reduce fibrosis caused by multiple upstream stimuli.

In conclusion, the present study is the first to demonstrate the potential role of CRLF1 in HLF formation. CRLF1 may act as an independent regulator to promote the pathogenesis of HLF formation via ERK signaling pathway at the post-transcriptional level. Up-regulation of CRLF1 in LF cells is the dominant transcriptional response to TGF-β1 and suppression of CRLF1 expression can obviously decrease HLF formation. Our findings identified CRLF1 as a key regulator in the pathogenesis of HLF (Figure 9), which offers potential strategies for the prevention and treatment of LSS.

## Data Availability Statement

The raw data supporting the conclusions of this article will be made available by the authors, without undue reservation, to any qualified researcher.

## Ethics Statement

The studies involving human participants were reviewed and approved by The Institutional Ethics Review Committee of the Third Affiliated Hospital of Southern Medical University. The patients/participants provided their written informed consent to participate in this study. The animal study was reviewed and approved by Ethics Committee for Animal Research of Southern Medical University.

## Author Contributions

LW and ZMZ contributed to the design of the work, analysis, interpretation, and manuscript writing. ZYZ, XA, and PL contributed to the data acquisition. ZL and TJ contributed to the data analysis and interpretation. All authors approved the submitted version and agreed to be personally accountable for the authors own contributions and manuscript contents.

## Conflict of Interest

The authors declare that the research was conducted in the absence of any commercial or financial relationships that could be construed as a potential conflict of interest.

## Footnotes

1 http://www.ncbi.nlm.nih.gov/geo/
