# Supplementary material for: CRLF1 Is a Key Regulator in the Ligamentum Flavum Hypertrophy
Source: Front Cell Dev Biol. 2020 Sep 18;8:858. doi: 10.3389/fcell.2020.00858 (PMC7533558; doi:10.3389/fcell.2020.00858)
Supplement: Supplementary file 1 [file Data_Sheet_1.DOCX]

Supplementary Material

**Supplementary** **Table 1.** General data of patients. Independent-sample t-test; P < 0.05 is considered to be significant.

|  | Non-HLF（n = 20） | HLF（n = 20） | P Value |
| --- | --- | --- | --- |
| Age | 62.9 ± 1.8 | 65± 2.2 | 0.4 |
| Gender  Lumbar level | 10 male，10 female  L_4-5_ | 10 male，10 female  L_4-5_ |  |
| LF thickness | 2.23 ± 0.11 mm | 5.29 ± 0.14mm | <0.0001 |

**Supplementary Table 2.** Primers used for quantitative reverse transcription polymerase chain reaction (human).

| Gene Name |  |  |
| --- | --- | --- |
| COL1A1 | Forward | 5’-GAGGGCCAAGACGAAGACATC-3’ |
|  | Reverse | 5’-CAGATCACGTCATCGCACAAC-3’ |
| COL3A1 | Forward | 5’-GCCAAATATGTGTCTGTGACTCA-3’ |
|  | Reverse | 5’-GGGCGAGTAGGAGCAGTTG-3’ |
| MMP2 | Forward | 5’- ATTGTATTTGATGGCATCGCTC -3’ |
|  | Reverse | 5’- ATTCATTCCCTGCAAAGAACAC -3’ |
| a-SMA | Forward | 5′-CCAGAGCCATTGTCACACAC-3′ |
|  | Reverse | 5′-CAGCCAAGCACTGTCAGG-3′ |
| CRLF1 | Forward | 5’-GATGCAGAAGTCGCACAAG-3’ |
|  | Reverse | 5’-TTATCTGGCAGGACCTCTCG -3’ |
| CLCF1 | Forward | 5’- CTGGGACCTATCTGAACTACCT -3’ |
|  | Reverse | 5’- CAGTTTGTCATTGAGGCTTCG -3’ |
| GAPDH | Forward | 5’-TGTTCGTCATGGGTGTGAAC-3’ |
|  | Reverse | 5’-ATGGCATGGACTGTGGTCAT-3’ |

COL1A1 indicates collagen type 1 alpha 1; COL3A1, collagen type 3 alpha 1; MMP-2, matrix metalloproteinase-2; a-SMA, Actin Alpha 2, Smooth Muscle; GAPDH, glyceraldehyde-3-phosphate dehydrogenase.

**Supplementary Table 3.** CRLF1-targeting siRNA sequences of human and mouse.

| Human |  |  |
| --- | --- | --- |
| RNAi 1# | Sense | 5’-GGAUGCAGAAGUCGCACAATT-3’ |
|  | Antisense | 5’-UUGUGCGACUUCUGCAUCCTT-3’ |
| RNAi 2# | Sense | 5’-GCCAAAUACCAGAUCCGCUTT-3’ |
|  | Antisense | 5’-AGCGGAUCUGGUAUUUGGCTT-3’ |
| RNAi 3 # | Sense | 5’-GGCUCUCUUUACGCCCUAUTT-3’ |
|  | Antisense | 5’- AUAGGGCGUAAAGAGAGCCTT-3’ |
| Mouse |  |  |
| RNAi 1# | Sense | 5’-UGGCUCAAGAAGCACGCAU-3’ |
|  | Antisense | 5′-AUGCGUGCUUCUUGAGCCA-3′ |
| RNAi 2# | Sense | 5′-GAUCUGAUGUCCUCACACU-3′ |
|  | Antisense | 5’-AGUGUGAGGACAUCAG-3’ |
| RNAi 3# | Sense | 5’-UCCUCUUCCAAGCCAAGUA-3’ |
|  | Antisense | 5’-UACUUGGCUUGGAAGAGGA-3’ |

**Supplementary Table 4.** Analysis of RNAseq in GEO datasets identified 197 upregulated genes.

| GENE_SYMBOL | logFC | P.Value | GENE_SYMBOL | logFC | P.Value |
| --- | --- | --- | --- | --- | --- |
| AMTN | 6.69 | 0.001141 | PDE4D | 2.78 | 0.001298 |
| CA9 | 4.62 | 0.000759 | SHOX2 | 2.76 | 0.000186 |
| GABRD | 4.46 | 1.63E-05 | CILP2 | 2.76 | 0.001618 |
| COL2A1 | 4.33 | 0.000224 | SEZ6L | 2.73 | 0.00102 |
| LY6D | 4.22 | 0.000216 | PAQR5 | 2.73 | 0.003969 |
| RN5-8S1 | 4.12 | 0.001919 | RIMS1 | 2.69 | 0.001651 |
| MSR1 | 4.08 | 0.001751 | HHIPL2 | 2.69 | 0.005587 |
| CLEC3A | 3.9 | 0.000943 | GDF6 | 2.68 | 0.005521 |
| SOX11 | 3.77 | 0.001304 | COL11A2 | 2.67 | 3.3E-05 |
| CHRDL2 | 3.76 | 0.000282 | PLOD1 | 2.67 | 0.000646 |
| SBSN | 3.75 | 0.000558 | IRX4 | 2.67 | 0.056244 |
| CCL3 | 3.67 | 0.000609 | PCDH11Y | 2.67 | 0.070166 |
| MMP1 | 3.66 | 0.004165 | IGFBP1 | 2.67 | 0.097309 |
| CYTL1 | 3.55 | 0.000617 | ACAN | 2.66 | 0.000521 |
| TREM2 | 3.53 | 0.000881 | TAS2R13 | 2.66 | 0.00024 |
| CLEC18C | 3.53 | 0.002697 | COL10A1 | 2.66 | 0.007319 |
| PVRL4 | 3.52 | 0.000489 | MMP13 | 2.64 | 0.081917 |
| CRLF1 | 3.47 | 0.007139 | SLC39A14 | 2.63 | 0.00043 |
| C10orf81 | 3.38 | 0.00482 | MFI2 | 2.62 | 5.99E-05 |
| IL11 | 3.35 | 0.000336 | PPAPDC1A | 2.62 | 0.0289 |
| TREM1 | 3.29 | 0.001716 | C2orf72 | 2.6 | 0.000552 |
| CCL3L3 | 3.26 | 0.000755 | SPP1 | 2.58 | 0.021462 |
| NXPH4 | 3.24 | 0.000536 | KIAA1199 | 2.58 | 0.056283 |
| PAX6 | 3.17 | 0.042559 | FGFR3 | 2.57 | 0.000396 |
| CYP27C1 | 3.16 | 0.000667 | GRIK3 | 2.57 | 0.010411 |
| LINC00473 | 3.16 | 0.009366 | CXCL3 | 2.57 | 0.056564 |
| FN1 | 3.11 | 0.000561 | KCNJ5 | 2.56 | 0.014392 |
| LOC100499467 | 3.11 | 0.001005 | ZMAT4 | 2.56 | 0.039934 |
| CCL4 | 3.08 | 0.001129 | TPSD1 | 2.54 | 0.005336 |
| ULBP1 | 3.08 | 1.25E-05 | CST1 | 2.54 | 0.054105 |
| LOC150622 | 3.07 | 0.002949 | GREM1 | 2.52 | 3.53E-05 |
| KIAA1751 | 3.06 | 0.00013 | LOC100129112 | 2.52 | 0.005533 |
| CCL18 | 3.06 | 0.002585 | IL21R | 2.51 | 0.001175 |
| CBLN4 | 3.06 | 0.025151 | FCGBP | 2.51 | 0.011024 |
| CRTAC1 | 3.04 | 0.000835 | GLYATL1 | 2.5 | 0.002153 |
| TNFSF11 | 3.03 | 0.000971 | TPSAB1 | 2.5 | 0.003204 |
| SYT13 | 3 | 0.005585 | PART1 | 2.5 | 0.003329 |
| COL9A2 | 2.97 | 3.77E-05 | POSTN | 2.5 | 0.005266 |
| C10orf105 | 2.92 | 0.001637 | KBTBD13 | 2.49 | 0.00247 |
| TUBB3 | 2.92 | 0.014425 | APBA1 | 2.47 | 9.3E-05 |
| SDS | 2.9 | 0.003158 | FAM84A | 2.47 | 0.006263 |
| MGC4294 | 2.89 | 0.000788 | CLEC7A | 2.47 | 0.006632 |
| STMN2 | 2.88 | 0.001171 | HMOX1 | 2.46 | 0.006882 |

| GENE_SYMBOL | logFC | P.Value | GENE_SYMBOL | logFC | P.Value |
| --- | --- | --- | --- | --- | --- |
| TMEM59L | 2.45 | 0.000403 | P4HA3 | 2.26 | 0.023056 |
| FAM106CP | 2.45 | 0.036818 | CHRM3 | 2.25 | 3.18E-06 |
| XLOC_001851 | 2.44 | 0.003832 | CGREF1 | 2.22 | 6.17E-05 |
| IGSF21 | 2.44 | 8.17E-05 | FAM176A | 2.22 | 0.000105 |
| GPR64 | 2.44 | 0.002885 | CD70 | 2.22 | 0.002753 |
| CPZ | 2.44 | 0.007419 | MT1H | 2.21 | 4.13E-05 |
| LRRC8E | 2.44 | 0.01214 | STEAP3 | 2.21 | 0.000179 |
| KIR2DS2 | 2.43 | 0.0944 | TNFRSF19 | 2.21 | 0.003194 |
| HAPLN1 | 2.42 | 0.000109 | LCN10 | 2.21 | 0.003372 |
| SCRG1 | 2.42 | 0.000256 | SLC16A10 | 2.21 | 0.004302 |
| DKK1 | 2.41 | 0.004939 | ADORA3 | 2.21 | 0.004315 |
| WISP3 | 2.41 | 0.008713 | ADAMTS2 | 2.2 | 0.000115 |
| CLEC18B | 2.41 | 0.019577 | LRRC15 | 2.17 | 0.001964 |
| MIA | 2.4 | 0.000651 | TRPV4 | 2.17 | 0.004346 |
| COL9A3 | 2.4 | 0.001025 | LECT1 | 2.17 | 0.009064 |
| KLF6 | 2.4 | 0.003105 | ASPHD1 | 2.17 | 0.00915 |
| STON1-GTF2A1L | 2.39 | 0.007113 | CILP | 2.17 | 0.031196 |
| ERVMER34-1 | 2.38 | 0.000129 | SLC12A1 | 2.17 | 0.071824 |
| LOC283867 | 2.38 | 0.010906 | ADAMTS6 | 2.16 | 0.000353 |
| CACNA1I | 2.38 | 0.018938 | CD300C | 2.16 | 0.007983 |
| SEZ6L2 | 2.37 | 0.011435 | MAGED4B | 2.16 | 0.009694 |
| CST2 | 2.37 | 0.014579 | BGN | 2.14 | 8.62E-05 |
| KCNN4 | 2.36 | 0.000672 | PENK | 2.14 | 0.001031 |
| CCK | 2.35 | 0.024376 | BMP1 | 2.14 | 0.00187 |
| SEMA7A | 2.35 | 0.029325 | FCGR1B | 2.14 | 0.002526 |
| CLVS2 | 2.34 | 0.010026 | SERPINA5 | 2.14 | 0.007869 |
| APLN | 2.33 | 0.002404 | ARSI | 2.14 | 0.023623 |
| XLOC_005770 | 2.32 | 4.54E-05 | SLC16A3 | 2.13 | 0.000332 |
| ITIH6 | 2.32 | 0.001612 | BMPR1B | 2.12 | 1.29E-06 |
| SRMS | 2.32 | 0.052428 | GALNT5 | 2.12 | 0.006311 |
| RASSF10 | 2.32 | 0.087766 | SERPINE2 | 2.11 | 0.002049 |
| GLT25D2 | 2.31 | 0.000171 | THY1 | 2.11 | 0.007319 |
| PLXDC1 | 2.31 | 0.007684 | FLJ46906 | 2.09 | 0.000188 |
| THBS1 | 2.3 | 2E-05 | CLLU1 | 2.09 | 0.000389 |
| ANGPTL6 | 2.29 | 0.002028 | SPINT1 | 2.09 | 0.001107 |
| SLAMF8 | 2.29 | 0.002724 | EFHC2 | 2.09 | 0.010623 |
| CHI3L2 | 2.28 | 0.056356 | XLOC_006037 | 2.09 | 0.024906 |
| SLC37A2 | 2.27 | 0.008786 | FBLN7 | 2.08 | 0.000323 |
| NGEF | 2.27 | 0.005139 | GPR98 | 2.08 | 0.055532 |
| FPR3 | 2.27 | 0.008342 | LOC339240 | 2.07 | 0.01127 |
| PAMR1 | 2.26 | 0.000692 | TM7SF4 | 2.06 | 0.004091 |
| SMOC2 | 2.26 | 0.000943 | ADAM12 | 2.06 | 0.013866 |
| C2orf82 | 2.26 | 0.002225 | FGFBP2 | 2.06 | 0.017887 |

| GENE_SYMBOL | logFC | P.Value | GENE_SYMBOL | logFC | P.Value |
| --- | --- | --- | --- | --- | --- |
| IL28RA | 2.05 | 0.001551 | LOR | 2 | 0.0037 |
| NELL1 | 2.05 | 0.013325 | DKK3 | 2.2 | 0.000155 |
| SNORA2A | 2.04 | 0.003832 | DLX4 | 2.2 | 0.001211 |
| FOLH1 | 2.04 | 0.007535 | KIAA1324 | 2.2 | 0.043354 |
| TMEM2 | 2.04 | 0.00118 | KLK1 | 2.2 | 0.066443 |
| TNFAIP6 | 2.03 | 0.004953 | LOC642422 | 2.19 | 0.10268 |
| DIO2 | 2.03 | 0.005646 | PCDH11X | 2.18 | 0.038077 |
| LOC100130111 | 2.03 | 0.00678 | LPAR5 | 2.17 | 0.000172 |
| SERPINA3 | 2.02 | 0.006597 | MMP7 | 2 | 0.010162 |
| PYCR1 | 2.02 | 0.004151 | CD84 | 2 | 0.023266 |
| FAP | 2.01 | 0.003109 | AQP8 | 2 | 0.035914 |
| SCG5 | 2.01 | 0.003511 | SLC6A7 | 2 | 0.060299 |
| ACP5 | 2.01 | 0.026459 |  |  |  |

**Supplementary Table 5.** Analysis of iTRAQ data identified 127 upregulated genes.

| GENE_SYMBOL | AVG. | T.TEST | GENE_SYMBOL | AVG. | T.TEST |
| --- | --- | --- | --- | --- | --- |
| CLU | 30.33346 | 2.2E-09 | CRTAC1 | 3.261481 | 2.09E-05 |
| PRG4 | 25.51819 | 3.3E-07 | KRT9 | 3.098109 | 0.000191 |
| CRLF1 | 23.68616 | 3E-09 | MAMDC2 | 3.089426 | 8.72E-08 |
| APCS | 20.0204 | 5.19E-08 | RHOC | 3.071121 | 1.05E-06 |
| APOB | 19.58365 | 1.23E-08 | BLVRB | 3.040348 | 0.001405 |
| TNFRSF11B | 19.24022 | 4.85E-07 | SAA4 | 3.039253 | 1.42E-06 |
| HTRA1 | 17.31465 | 7.18E-06 | COL1A2 | 3.012655 | 0.001697 |
| CILP | 16.8814 | 1.02E-05 | C4BPA | 2.990617 | 9.6E-05 |
| APOE | 12.70924 | 7.47E-07 | ITIH2 | 2.858556 | 2.85E-06 |
| MFGE8 | 11.12859 | 7.56E-05 | RNASE4 | 2.845368 | 0.000781 |
| CA1 | 10.90193 | 2.14E-05 | ITGAV | 2.761041 | 5.02E-05 |
| LYZ | 10.67871 | 1.29E-06 | PPIC | 2.739444 | 0.000299 |
| SRPX2 | 9.811407 | 1.4E-07 | HRG | 2.739127 | 7.36E-08 |
| THBS1 | 9.708624 | 1.42E-06 | CYCS | 2.728488 | 4E-06 |
| SERPINE2 | 8.779501 | 0.00014 | APOC1 | 2.705299 | 3.99E-06 |
| APOA4 | 8.605912 | 3.61E-08 | APOC3 | 2.62564 | 2.47E-06 |
| THBS4 | 8.290345 | 3.88E-05 | AK1 | 2.590109 | 0.00064 |
| ACAN | 7.814309 | 0.000121 | CP | 2.585625 | 8.48E-05 |
| MMP3 | 7.015802 | 8.03E-06 | PSAP | 2.574102 | 1.12E-06 |
| VTN | 6.976948 | 2.68E-07 | CD63 | 2.554027 | 2.63E-05 |
| APOA1 | 6.936471 | 0.000248 | PTX3 | 2.527559 | 8.43E-05 |
| CHAD | 6.803079 | 5.17E-06 | IDH2 | 2.497907 | 0.000835 |
| CAT | 6.723746 | 2.5E-06 | PRDX6 | 2.48519 | 8.05E-07 |
| TIMP3 | 6.528113 | 2.97E-07 | GNAI2 | 2.455584 | 2.7E-06 |
| PRDX2 | 6.330278 | 1.25E-06 | TYMP | 2.437799 | 0.001396 |
| PCOLCE2 | 6.064633 | 5.99E-06 | APRT | 2.415127 | 5.56E-06 |
| KRT13 | 5.938882 | 1.25E-07 | CD47 | 2.391136 | 5.59E-06 |
| HAPLN1 | 5.825963 | 8.7E-07 | IGHV3-49 | 2.284524 | 0.002014 |
| COL8A1 | 5.762478 | 1.31E-05 | ITIH4 | 2.274715 | 1.11E-05 |
| C9 | 5.676178 | 1.42E-06 | C6 | 2.246198 | 2.14E-07 |
| CD9 | 5.516056 | 1.08E-06 | CPB2 | 2.239499 | 6.92E-06 |
| CCDC80 | 5.501574 | 8.63E-08 | ANXA5 | 2.235346 | 0.001593 |
| SOD2 | 5.123954 | 1.24E-05 | IGHA2 | 2.226181 | 1.76E-05 |
| QSOX1 | 5.115834 | 1.5E-05 | SOST | 2.201074 | 0.002245 |
| A2M | 4.565623 | 4.31E-06 | APOC2 | 2.113242 | 4.34E-07 |
| HP | 4.114016 | 8.87E-05 | LDHA | 2.079103 | 0.00045 |
| FGFBP2 | 4.100846 | 3.47E-09 | UBA52 | 2.072996 | 0.002619 |
| C3 | 4.086165 | 9.12E-05 | SLPI | 2.043378 | 0.000582 |
| CA2 | 4.05094 | 5.2E-06 | FTL | 2.043347 | 0.001764 |
| FTH1 | 3.889172 | 0.000688 | PON1 | 2.022167 | 1.93E-05 |
| COMP | 3.839188 | 0.000234 | ANG | 2.016556 | 8.89E-05 |
| EZR | 3.707431 | 2.08E-07 | ANXA4 | 1.997101 | 1.16E-05 |
| VCAN | 3.614318 | 0.000888 | PNP | 1.974183 | 0.004413 |

| GENE_SYMBOL | AVG. | T.TEST | GENE_SYMBOL | AVG. | T.TEST |
| --- | --- | --- | --- | --- | --- |
| S100A1 | 1.973377 | 6.56E-05 | LBP | 1.66191 | 0.001849 |
| BPGM | 1.95377 | 5.27E-07 | GSTO1 | 1.649683 | 0.000225 |
| RRAS | 1.944361 | 3.52E-05 | EEF1A1P5 | 1.649338 | 0.017232 |
| PLG | 1.914407 | 0.004054 | AFM | 1.637437 | 0.001307 |
| MYADM | 1.912566 | 0.023209 | PARK7 | 1.626069 | 0.006074 |
| SLC4A1 | 1.905874 | 0.005951 | F13A1 | 1.624229 | 0.018091 |
| CTSD | 1.893115 | 0.001718 | KRT2 | 1.612575 | 0.000123 |
| CAPS | 1.879688 | 0.000107 | MSN | 1.605229 | 0.000551 |
| PFKL | 1.866184 | 0.001809 | CFHR1 | 1.604787 | 0.000207 |
| ITIH1 | 1.861446 | 0.000134 | MYO1D | 1.604774 | 2.23E-08 |
| ALAD | 1.858616 | 2.13E-06 | PGD | 1.592912 | 0.000158 |
| TPBG | 1.858569 | 9.77E-05 | FAM129B | 1.590606 | 0.013271 |
| S100A8 | 1.8359 | 0.000119 | C7 | 1.570523 | 0.003318 |
| SERPING1 | 1.829434 | 0.000182 | SLC25A5 | 1.568638 | 0.000779 |
| STAB1 | 1.737917 | 7.88E-06 | CD55 | 1.557582 | 2.84E-07 |
| COL2A1 | 1.724635 | 0.000884 | APOM | 1.553389 | 0.000103 |
| S100A9 | 1.714085 | 0.000124 | COL1A1 | 1.548865 | 6.51E-06 |
| CRYAB | 1.70114 | 0.011819 | SEMA3B | 1.537245 | 0.001958 |
| ANXA1 | 1.694522 | 7.21E-06 | PLCD1 | 1.520713 | 1.43E-07 |
| RAN | 1.694234 | 0.00014 | ATP2B4 | 1.51152 | 0.026502 |
| COL3A1 | 1.689009 | 0.035123 |  |  |  |
|  |  |  |  |  |  |
|  |  |  |  |  |  |
|  |  |  |  |  |  |
|  |  |  |  |  |  |
